# Structural Basis for PPARs Activation by The Dual PPARα/γ Agonist Sanguinarine: A Unique Mode of Ligand Recognition

**DOI:** 10.3390/molecules26196012

**Published:** 2021-10-03

**Authors:** Siyu Tian, Rui Wang, Shuming Chen, Jialing He, Weili Zheng, Yong Li

**Affiliations:** The State Key Laboratory of Cellular Stress Biology, Innovation Center for Cell Signaling Network, School of Life Sciences, Xiamen University, Xiamen 361005, China; tiansy12@lzu.edu.cn (S.T.); wangrui@163.com (R.W.); 21620152202894@stu.xmu.edu.cn (S.C.); hjl_lingfeng@163.com (J.H.); zwldlaiyita@163.com (W.Z.)

**Keywords:** dual agonist, crystal structure, pharmacophore, nuclear receptors, peroxisome proliferator-activated receptors

## Abstract

Peroxisome proliferator-activated receptors (PPARs) play crucial roles in glucose and lipid metabolism and inflammation. Sanguinarine is a natural product that is isolated from Sanguinaria Canadensis, a potential therapeutic agent for intervention in chronic diseases. In this study, biochemical and cell-based promoter-reporter gene assays revealed that sanguinarine activated both PPARα and PPARγ, and enhanced their transcriptional activity; thus, sanguinarine was identified as a dual agonist of PPARα/γ. Similar to fenofibrate, sanguinarine upregulates the expression of PPARα-target genes in hepatocytes. Sanguinarine also modulates the expression of key PPARγ-target genes and promotes adipocyte differentiation, but with a lower adipogenic activity compared with rosiglitazone. We report the crystal structure of sanguinarine bound to PPARα, which reveals a unique ligand-binding mode of sanguinarine, dissimilar to the classic Y-shaped binding pocket, which may represent a new pharmacophore that can be optimized for selectively targeting PPARα. Further structural and functional studies uncover the molecular basis for the selectivity of sanguinarine toward PPARα/γ among all three PPARs. In summary, our study identifies a PPARα/γ dual agonist with a unique ligand-binding mode, and provides a promising and viable novel template for the design of dual-targeting PPARs ligands.

## 1. Introduction

Peroxisome proliferator-activated receptors (PPARs) are ligand-activated transcription factors that belong to the nuclear receptor superfamily involved in lipid and glucose metabolism, adipogenesis, and inflammation [1]. Three different isoforms, namely PPARα, PPARδ (also known as PPARβ), and PPARγ, share conserved structural components comprised of the N-terminal–A/B domain that harbors ligand-independent activation function 1 (AF-1), the DNA-binding domain (DBD), a hinge region, and the C-terminal ligand-binding domain (LBD) that contains the ligand-dependent activation function 2 (AF2) (Figure S1A) [2,3]. Ligand-regulated physiological actions are mainly mediated through the LBDs, which bind to ligands and then recruit nuclear receptor co-activators or co-repressors to regulate the expression of downstream targeted genes [4,5].

PPARs have been recognized as therapeutic targets for the treatment of diabetes, dyslipidemia, and nonalcoholic steatohepatitis, or fatty liver disease (NASH/NAFLD) [6]. While PPARα agonist fibrates are well-tolerated by most patients, some adverse effects have been reported, such as gastrointestinal disease, sleep disorder, renal disease, and liver dysfunction [7,8]. Thiazolidinediones (TZDs) have a high affinity and a full agonism to PPARγ. Essentially, they improve insulin sensitivity and are used clinically for the treatment of T2DM, which is associated with various metabolic disorders, including obesity, hypertension, and dyslipidemia. Nevertheless, undesirable effects can occur during the continuous use of TZDs, such as weight gain, fluid retention, bone fractures, and an increased rate of heart failure [9,10,11]. Consequently, a new drug design strategy for PPAR ligands, distinct from fibrates and TZDs, may yield more efficacious, PPAR-targeted drugs with fewer adverse effects.

Sanguinarine, a type of quaternary benzophenanthridine alkaloid (QBA) and regarded as a “secondary metabolite” or “natural product”, is mainly extracted from the roots of Sanguinaria canadensis (blood root), Chelidonium majus (greater celandine), or other medicinal poppy fumaria species [12,13]. Due to its remarkable biological activities and chemical structure, sanguinarine has attracted extensive attention. Previously, it has been shown that sanguinarine plays an important role in chronic diseases, such as type 2 diabetes, cancer, etc. [14,15]. Sanguinarine exhibits anti-cancer potential by inducing apoptosis and/or anti-proliferative effects on tumor cells by targeting ERK1/2, caspase, Bcl-2, and STAT3. Sanguinarine also shows anti-angiogenic and anti-invasive properties by regulating VEGF, NF-κB, and MMP [12,16]. In addition, the therapeutic potentials of sanguinarine regarding cardiovascular disease, including anti-platelet and positive inotropic effects, have been reported [17]. Sanguinarine further has a promising anti-hypertensive effect, interferes with the renin–angiotensin system and possibly other blood-pressure-regulating pathways, and induces calcium mobilization, thromboxane, and cAMP production [14,18]. However, the molecular target of sanguinarine remains unclear.

In this study, biochemical and cell-based promoter-reporter gene assays reveal that sanguinarine activates both PPARα and PPARγ, and enhances their transcriptional activity as a dual PPARα/γ agonist (Figure S1B). Moreover, sanguinarine modulates the key PPARα-target gene expression in hepatocytes and key PPARγ-target gene expression in adipocyte differentiation, but shows a lower adipogenic activity than TZDs. We solved the crystal structure of sanguinarine-bound PPARα, which revealed a novel and unique ligand-binding mode of sanguinarine that differs from the classic Y-shaped binding pocket [19,20]. Through a combination of mutagenesis, biochemical binding studies, and structural analysis, we uncovered the molecular basis for the selectivity of sanguinarine toward PPARα/γ among all three PPARs. Consequently, our study may represent a unique scaffold for the design of dual-targeting PPARs agonists.

## 2. Results

### 2.1. Identification of Sanguinarine as a Dual PPARα/γ Agonist

**Figure 1:**
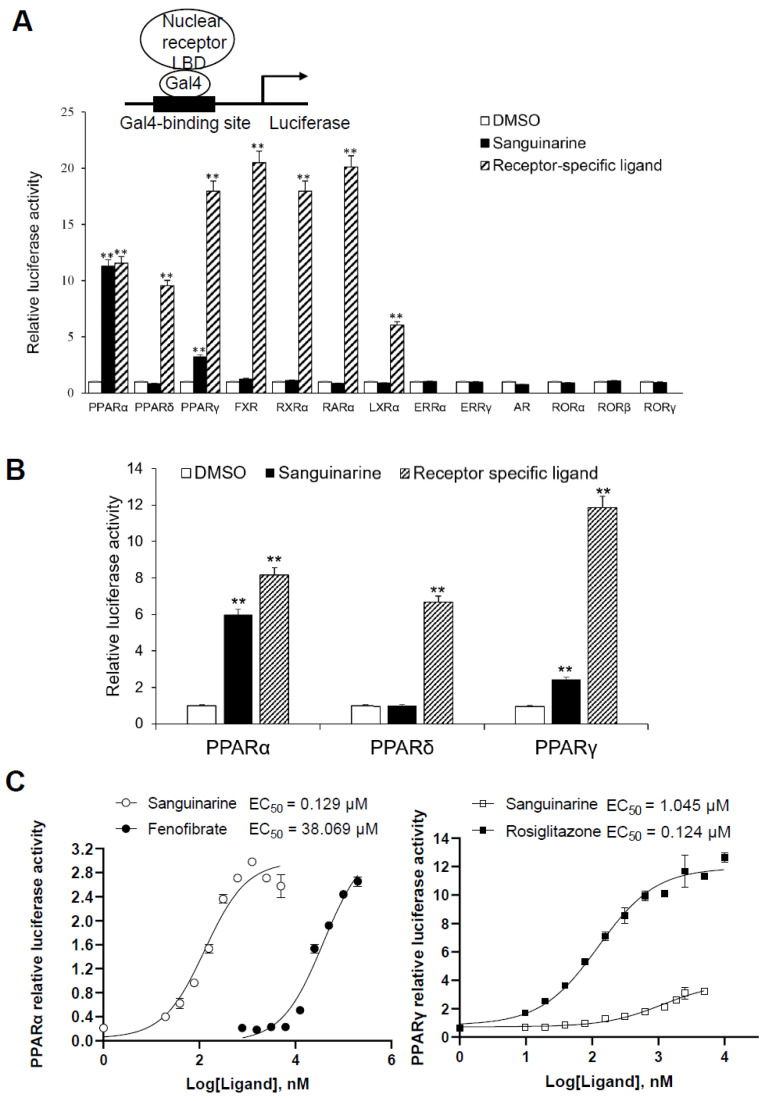
Identification of Sanguinarine as a dual PPAR α/γ agonist. (**A**) Receptor-specific transactivation by sanguinarine. 293T cells were co-transfected with pG5Luc reporter plasmid, together with the plasmids encoding various NR–LBDs fused with the Gal4–DNA-binding domain. After transfection, cells were treated with DMSO (white bars), 1 μM sanguinarine (black bars), or ligands specific to each receptor (striped bars): PPAR α, 1 μM GW590735; PPAR δ, 1 μM GW0472; PPAR γ, 1 μM rosiglitazone; FXR, 0.1 μM GW4064; RXR α, 1 μM 9 cis RA; RAR α, 1 μM ATRA; LXR α,1 μM T0901317. Luciferase activity is reported as normalized to renilla activity. For clarity, ERR, estrogen-related receptor; AR, androgen receptor; ROR, retinoid-related orphan receptor. (**B**) Sanguinarine increased the expression of PPAR α/γ native promoter luciferase reporter. 293T cells were co-transfected with plasmids encoding full-length PPARs and their response element. After transfection, cells were treated with DMSO (white bars), 1 μM sanguinarine (black bars), or ligands specific to each receptor (striped bars). (**C**) Dose responses of sanguinarine in transactivating PPAR α/γ. 293 T cells were co-transfected with plasmids encoding PPAR–LBDs fused with the Gal4–DNA-binding domain and pG5Luc reporter. After transfection, cells were treated with various concentrations of sanguinarine and specific ligands. The results equate to the average of experiments performed in triplicate, with error bars indicating SDs; ** *p* < 0.01, compared with vehicle.

While considering the limitations of current PPAR agonists, we conducted a high-throughput AlphaScreen™ assay to search for novel ligands with distinct properties; this is widely used for detecting ligand-dependent interactions between nuclear receptors and their co-activators [21,22]. We found that the natural benzophenanthridine alkaloid sanguinarine, from the Enzo Natural Compound Library, greatly enhances the interaction between PPARα/γ–LBD and their co-activators (Figure S2). To further probe the physiological role of sanguinarine on PPAR signaling, we first characterized the transcriptional properties of PPARs in response to sanguinarine. The 293T cells were co-transfected with GAL4-driven plasmids, encoding three PPAR subtypes and various other nuclear receptors, respectively. Interestingly, results indicated that sanguinarine had a strong agonist activity on PPARα, and a weak activity on PPARγ, although not on PPARδ and the other nuclear receptors that were tested (Figure 1A). While previous chemical composition-target-pathways-disease network analysis found that FXR is a potential target for sanguinarine in the regulation of fibrosis [23], our data suggest that sanguinarine does not directly activate FXR. Following this, 293T cells were co-transfected with plasmids encoding full-length PPARs and their response element, PPRE. In agreement with GAL4-driven reporter assay results, sanguinarine could significantly activate PPARα, but had weak activity on PPARγ, and no impact on PPARδ (Figure 1B). Furthermore, full-dose curves revealed that sanguinarine activated PPARα and PPARγ in a concentration-dependent manner, with an approximate EC_50_ of 129 nM and 1045 nM (Figure 1C), respectively, suggesting that sanguinarine is a potent PPARα/γ dual agonist.

**Figure 2:**
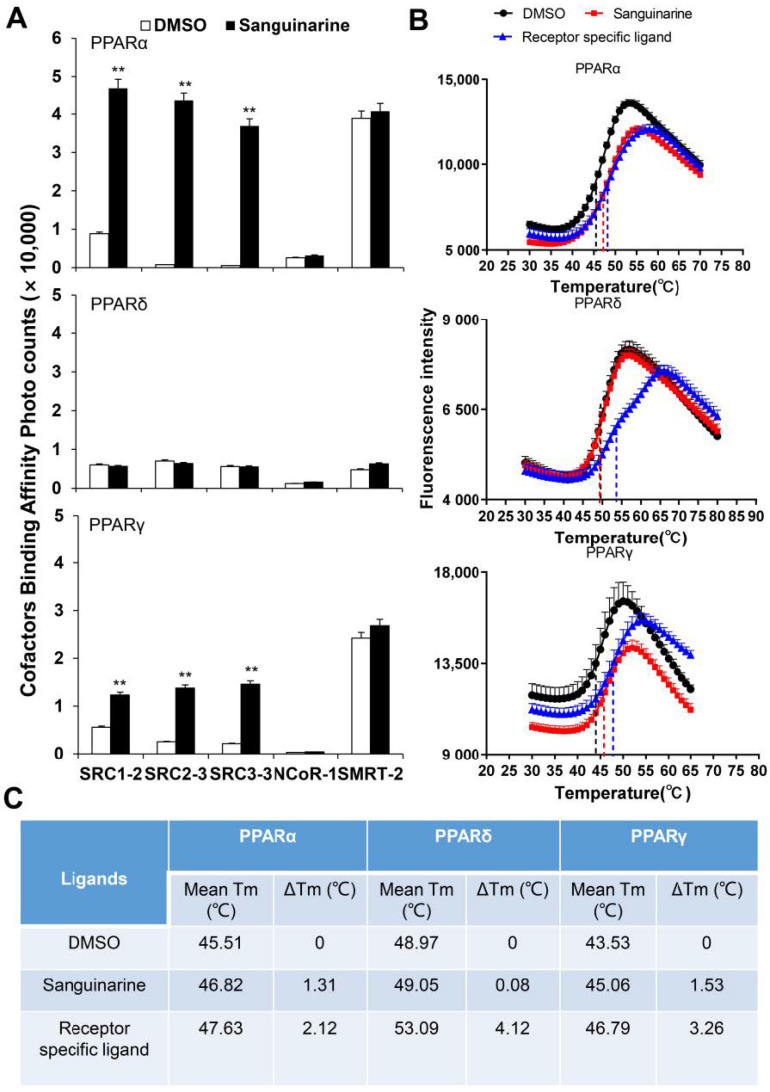
(**A**) Sanguinarine promotes the interaction of co-activator LXXLL motifs with PPARα, shown by AlphaScreen assays. This displays the interaction of PPARα, PPARδ, and PPARγ with various co-factor LXXLL motifs in response to 1 μM sanguinarine. Sanguinarine significantly improved the thermal stability of PPARα/γ. (**B**) Thermal shift assay fluorescence signals obtained for PPARs–LBD, with and without ligands. (**C**) Thermal stability characterization of the interactions between PPARs–LBD and ligands. The results equate to the average of experiments performed in triplicate, with error bars indicating SDs; ** *p* < 0.01, compared with vehicle.

PPARs regulate the activity of their targeted genes by recruiting co-activators, or by releasing co-repressors [24]. The agonists induce various co-factors to bind to PPARs–LBD. To unravel the biochemical mechanism of PPARα/γ that is activated by sanguinarine, we adopted AlphaScreen assay to determine the ability of sanguinarine for modulating co-factor motifs. This is a widely used assay to detect ligand-dependent interaction between nuclear receptors and their co-factors. As Figure 2A demonstrates, sanguinarine enhanced the interaction of PPARα/γ with various co-activator LXXLL motifs from SRC1-2, SRC2-3, and SRC3-3, but not with co-repressor motifs from NCoR1 and SMRT2. In contrast, sanguinarine had no effect on PPARδ for recruiting its co-activator motifs. Ligand binding usually improves the thermal stability of NRs via structural and dynamic changes of the protein fold [25,26]. The thermal shift assay (TSA) showed that sanguinarine increased the thermal stability of PPARα and PPARγ, but the Tm of PPARδ remained unchanged (Figure 2B,C). These results reaffirm that sanguinarine is a dual PPARα/γ agonist, and strongly suggest a direct binding of sanguinarine with PPARα.

### 2.2. Sanguinarine Regulates PPARα-Target Genes in Hepatocytes and PPARγ-Target Genes in Adipocytes

**Figure 3:**
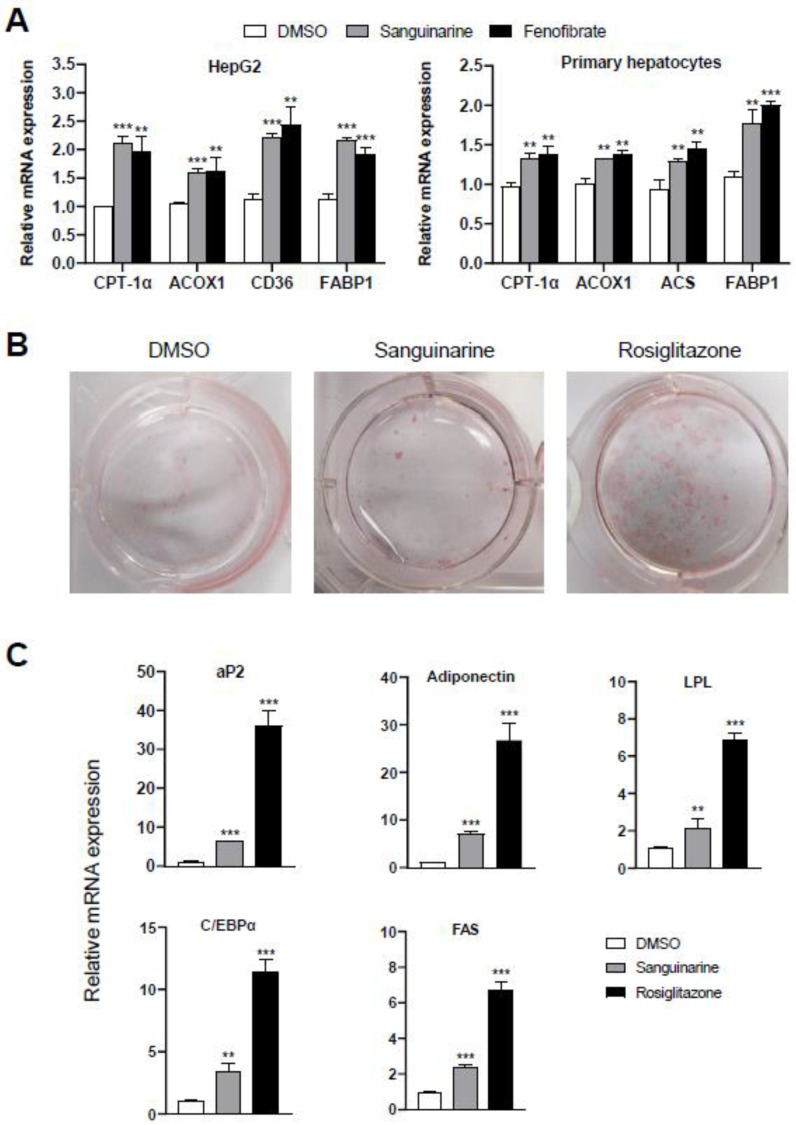
Effects of sanguinarine on PPARα/γ-target gene expression in cells. (**A**) Expression of PPARα-target genes. HepG2 cells and mouse primary hepatocytes were treated with either sanguinarine (1 µM) or fenofibrate (50 µM) for 48 h. (**B**) Oil red O staining of 3T3–L1 cells after treatment with 1 µM ligands was indicated for 7 days. (**C**) PPARγ-target gene expression during the adipocyte differentiation of 3T3–L1 cells induced by 1 µM ligands. The results equate to the average of experiments performed in triplicate, with error bars indicating SDs; ** *p* < 0.01, *** *p* < 0.01, compared with vehicle.

To further assess the modulation effects of sanguinarine on the targeted genes of PPARα/γ, we examined the mRNA expression level of PPARα-target genes in hepatocytes, and differentiation-dependent PPARγ-target genes in 3T3–L1 preadipocyte cells. Similar to fenofibrate, sanguinarine further upregulated the expression of PPARα-target genes in HepG2 cells and mouse primary hepatocytes, such as CPT-1α, ACOX1, CD36, ACS, and FABP1 (Figure 3A) [27]. Moreover, compared to rosiglitazone, sanguinarine induced a weakly adipocyte differentiation, as indicated by oil red O staining (Figure 3B). We additionally performed PPARγ-target gene analysis of 3T3–L1 cells treated with sanguinarine. As shown, the expression of differentiation-dependent PPARγ-target genes, such as aP2, LPL, adiponectin, C/EBPα, and FAS [28,29], was also induced by sanguinarine, but to a lesser extent than rosiglitazone (Figure 3C). Gene expression profile analysis is consistent with the results of oil red O staining. Thus, these results indicate that sanguinarine could regulate the expression of PPARα/γ-target genes in cells.

### 2.3. Overall Structure of Sanguinarine and PPARα with Unique Binding Mode

**Figure 4:**
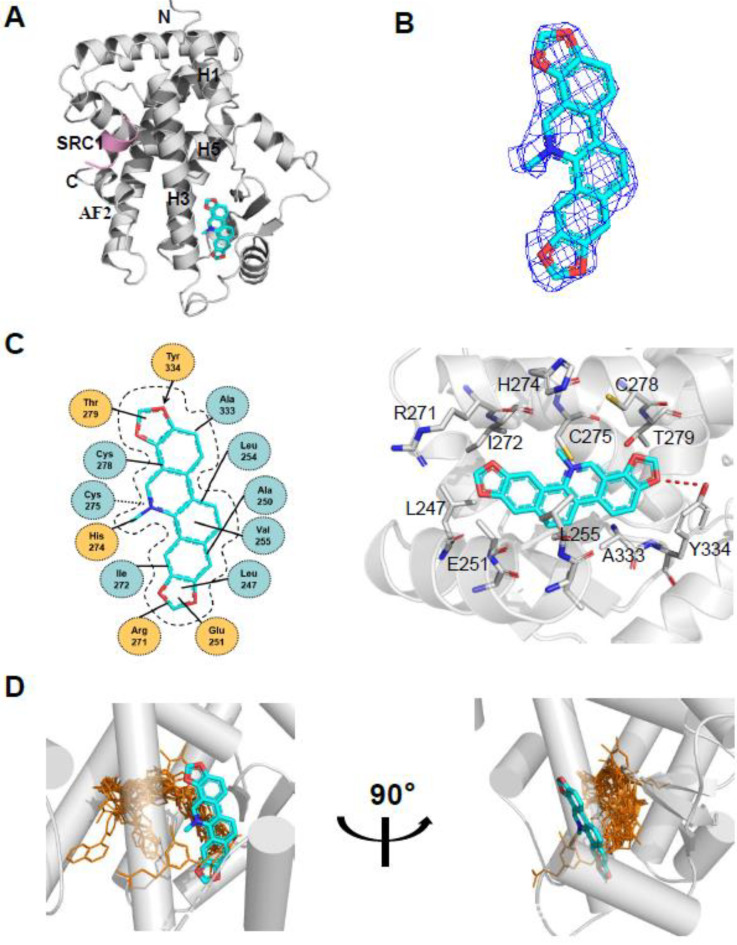
Structural analysis of the recognition of sanguinarine by PPARα. (**A**) The structure of sanguinarine bound with PPARα in cartoon representation. PPARα is colored in gray, and the SRC1-2 motif in pink. The bound sanguinarine is shown in stick representation with carbon, nitrogen, and oxygen atoms depicted in cyan, blue, and red, respectively. (**B**) A simulated-annealing 2Fo-Fc composite omit map (1.0 δ), showing the bound sanguinarine. (**C**) Key interactions of PPARα with sanguinarine. Red dashes represent hydrogen bond interaction. (**D**) Comparison of PPARα complexes. Superposition of reported PPARα complexes with agonists (PDB: 1I7G, 1K7L, 2NPA, 2P54, 2ZNN, 3ET1, 3FEI, 3G8I, 3KDU, 3SP6, 3VI8, 4BCR, 4CI4, 5AZT, 5HYK, 6KXX and 6L96) overlaid onto the PPARα complex with sanguinarine. Reported agonists are shown in gold lines, and sanguinarine is represented by cyan sticks.

To determine the molecular basis of the specific interaction with PPARα/γ, we solved the crystal structure of sanguinarine complexed with PPARα and SRC1-2 LXXLL motif (Table S1). Unfortunately, we were unable to calculate the crystal structure of sanguinarine complexed with PPARγ due to low-resolution x-ray diffraction. The structure revealed that the PPARα–LBD–sanguinarine complex was folded into a three-layer helical sandwich, with the C-terminal AF-2 helix positioned in a canonical active conformation, in agreement with the agonistic nature of the PPARα ligand (Figure 4A) [30,31]. The presence of sanguinarine was apparent in the highly revealing electron density map shown in Figure 4B. The binding of sanguinarine to PPARα was stabilized by a combination of hydrogen bonds, including Cys275 from helix 3, Arg271 from helix 3, Tyr334 from the loop between the β sheet, and hydrophobic interactions, such as Ala333 and Val255 (Figure 4C).

Surprisingly, compared with known PPARα Y-shaped ligand-binding pockets and ligand-binding sites, sanguinarine has a unique ligand-binding site in a novel PPARα ligand-binding pocket (Figure 4D), which may represent a new pharmacophore that can be optimized to selectively target PPARα.

### 2.4. Functional Correlation of the Sanguinarine-PPARα Interactions

**Figure 5:**
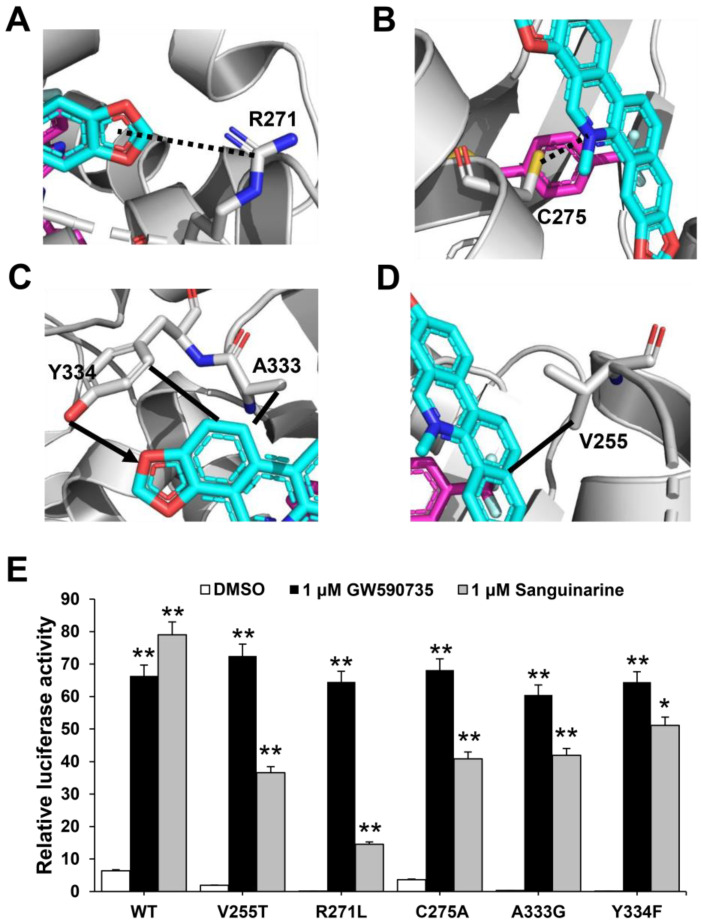
Functional correlation of the sanguinarine–PPARα interactions. (**A**–**D**) Molecular determinants of the interactions between sanguinarine ligand with PPARα. The bound sanguinarine is shown in stick representation, with carbon and oxygen atoms depicted in cyan and red, respectively. The PPARα ligand, GW735, resulted from superposition and is shown in stick representation with carbon, nitrogen, and oxygen atoms depicted in magenta, blue, and red, respectively. The hydrophobic interactions and hydrogen bonds are shown with lines and arrows, and the potential hydrogen bonds are indicated by dashed lines. (**E**) Effects of mutations of key PPARα residues on their transcriptional activity in response to sanguinarine treatment in cell-based reporter gene assays. 293T cells were co-transfected with plasmids encoding PPARα–LBD–WT or mutants, as indicated in the figures fused with the GAL4–DNA-binding domain together with the pG5Luc reporter. The cells were treated with 1 μM sanguinarine and 1 μM GW590735, respectively. The results equate to the average of experiments performed in triplicate, with error bars indicating SDs; * *p* < 0.05, ** *p* < 0.01, compared with vehicle.

To further verify the role of pocket residues in sanguinarine binding and PPARα activation, we mutated several key PPARα residues in contact with different groups of sanguinarine based on interaction forms and distance cut-off. We then tested the transcriptional activity of these mutated PPARα in response to sanguinarine in cell-based reporter assays using a GAL4-driven PPARα response reporter. The Arg271 and Cys275 are PPARα pocket residues that form van der Waals interactions with the O and N atoms of sanguinarine (Figure 5A,B). The R271L and C275A mutations decrease the activation of PPARα by sanguinarine, but not the PPARα-selective ligand GW735 in cell-based reporter assays using a GAL4-driven PPARα response reporter (Figure 5E). The hydrogen bond between the ligand and Tyr334 is critical for sanguinarine-bound PPARα, while this is not observed in the GW735-occupied receptor. As such, the Y334F mutation decreases the activation of PPARα by sanguinarine, but not GW735 (Figure 5C,E). In addition, the A333G and V255T mutations both decrease the activation of PPARα by sanguinarine, but not PPARα-selective ligand GW735 in cell-based assays (Figure 5C–E), which emphasizes the importance of these hydrophobic interactions for sanguinarine binding to PPARα. Collectively, these data affirm that the PPARα ligand-binding pocket has unique properties for differential binding of various ligands.

### 2.5. Conformational Changes of PPARα Induced by Sanguinarine Binding

**Figure 6:**
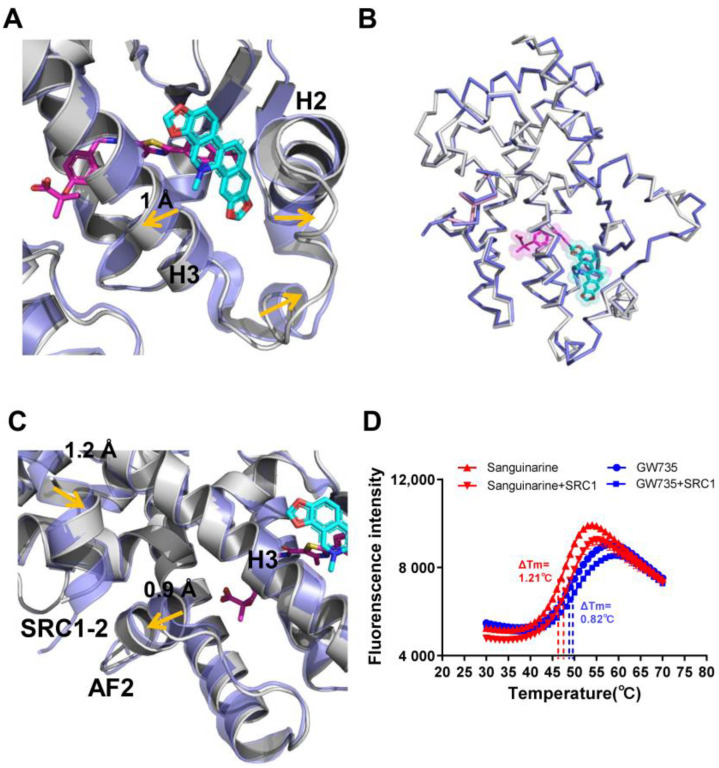
Conformational changes of PPARα induced by sanguinarine. (**A**–**C**) Superposition of the sanguinarine–PPARα structure (colored as in Figure 3) with the GW735–PPARα structure (PDB ID: 2P54; blue, where GW735 is magenta). The alignment reveals shifts for helix 2, helix 3, AF2, and SRC1, induced by sanguinarine binding which is indicated by arrows. (**D**) Thermal shift assay fluorescence signals obtained for PPARα/γ–LBD with ligands and the co-activator peptide, SRC1. The results are the average of experiments performed in triplicate, with error bars indicating SDs.

The conformational changes in sanguinarine binding with PPARα can be further clarified by the superposition of the sanguinarine–PPARα structure with the GW735–PPARα structure, which revealed that helix 2, helix 3 and the loop between H2 and H3, shifted outward significantly (Figure 6A), thereby increasing the affinity of PPARα with sanguinarine. Although the two PPARα structures are similar, with an RMSD of 0.438 Å (Figure 6B), their detailed topology remains somewhat different. Compared with the GW735–PPARα, the AF2 and helix 3 of the sanguinarine–PPARα shifted outward, which causes SRC1 to shift inward, closer to PPARα (Figure 6C). Furthermore, the thermal shift assay showed that SRC1 increased the thermal stability of sanguinarine–PPARα, compared with GW735–PPARα (Figure 6D). These results indicate the unique binding of sanguinarine to PPARα.

### 2.6. Molecular Determinants of PPAR Subtype Selectivity toward Sanguinarine

**Figure 7:**
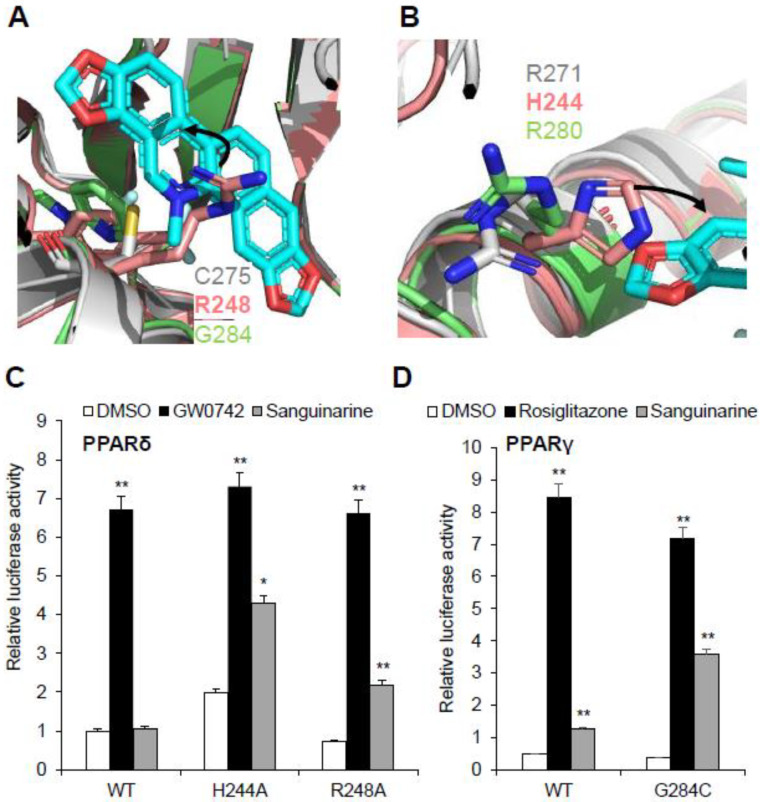
Structural basis of the sanguinarine binding specificity towards all three PPARs. (**A**,**B**) Superposition of PPARα–LBD (gray) with PPARδ–LBD (salmon, PDB code: 3KTM), and PPARγ–LBD (green, PDB code: 4EMA). Corresponding residues of PPARs are shown in stick representation. The amino acid steric hindrances are shown as curved arrows. (**C**,**D**) Effects of mutations of key PPARs residues on their transcriptional activity, in response to sanguinarine (black bars) in cell-based reporter gene assays. 293T cells were co-transfected with plasmids encoding full-length PPARs or PPARs mutants, as indicated together with a PPRE luciferase reporter. The cells were treated with 1 μM sanguinarine, and 1 μM GW0472 or 1 μM rosiglitazone, respectively. The results are the average of experiments performed in triplicate, with error bars indicating SDs; * *p* < 0.05, ** *p* < 0.01, compared with vehicle.

Sanguinarine has no impact on PPARδ activation, and this raises questions about the molecular basis of the ligand’s binding selectivity toward PPARα/γ. Notably, all three PPARs have high homology in both sequence and structure, such as the three-arm and Y-shaped ligand-binding pockets, and they have similar overall pocket size, but their detailed topology is clearly different (Figure S3) [3,32]. Structural comparison revealed a unique characteristic of the PPARδ ligand-binding pocket: that two key residues of the PPARδ–LBD, Arg248 and His244, are different from PPARα/γ, which impacts sanguinarine binding. It is quite evident that the side chains of residue Arg248 on helix 3 of PPARδ–LBD extend inward to generate a strong steric hindrance, and to occupy the binding position of sanguinarine (Figure 7A). As expected, sanguinarine activated the transcriptional activity of PPARδ–R248A mutation, in contrast to wild-type PPARδ (Figure 7C). Another key residue, His244 on helix 3 of PPARδ–LBD, is predicted to interfere with the sanguinarine binding by reducing the pocket size (Figure 7B). In the same manner, the H244A mutation enables PPARδ to respond to sanguinarine treatment (Figure 7C). There is also the question of why the binding of sanguinarine to PPARγ is weaker than that of PPARα. We contemplate that the thiol group of Cys275 is important for the binding of sanguinarine to PPARα. As expected, sanguinarine enhanced the transcriptional activity of the PPARγ–G284C mutation, compared to wild-type PPARγ. As such, structural and functional analysis revealed the molecular basis for the selectivity of sanguinarine binding to PPARα/γ, among all three PPARs.

## 3. Discussion

Sanguinarine is a quaternary benzophenanthridine alkaloid (QBA) found in many medicinal plants. It has been shown that sanguinarine plays an important role in chronic diseases, such as type 2 diabetes, cancer, etc. In our study, we found that sanguinarine is a modulating ligand for PPARα/γ by high-throughput AlphaScreen. Furthermore, we conducted a detailed analysis of the binding between sanguinarine and PPARs, via a combination of biochemical binding assays, mutagenesis, and structural analysis. The results from both biochemical and cell-based promoter-reporter gene assays showed that sanguinarine functions as a selective PPARα/γ dual agonist by recruiting co-activators and by activating the transcriptional activity of PPARα/γ.

The effort to develop PPAR agonists without any adverse effects led to the development of dual agonists (Figure S4). Although dual PPARα/γ agonist glitazars can lower plasma glucose and triglycerides, they have been associated with heart failure. The only glitazar still in clinical development is Saroglitazar. It has been approved in India for the treatment of diabetic dyslipidemia and hypertriglyceridemia with type 2 diabetes is not controlled by statin therapy, and NASH. Moreover, Saroglitazar has never been associated with cardiovascular complications. Previous studies have presented the high efficiency of dual PPARα/γ agonists against hyperglycemia and hyperlipidemia [33]. Therefore, we examined the mRNA expression level of PPARα-target genes in hepatocytes and differentiation-dependent PPARγ-target genes in 3T3–L1 preadipocyte cells. Similar to fenofibrate, sanguinarine upregulated the expression of PPARα-target genes in hepatocytes. Sanguinarine also modulated the expression of key PPARγ-target genes and promoted adipocyte differentiation, but with a lower adipogenic activity compared with rosiglitazone. Additionally, the therapeutic potential of sanguinarine in cardiovascular disease, including hypotensive, anti-platelet, and positive inotropic effects, have been reported [17]. The toxicity of Sanguinarine has also been reported in some studies [34], yet low and moderate doses of sanguinarine did not show a statistically significant cytotoxicity in human and porcine hepatocytes, and dose-dependent cytotoxicity was observed only at very high sanguinarine doses (25–100 µM) [35]. In addition, the acute oral LD_50_ in rats was reported as approximately 1658 mg/kg of sanguinarine. When provided through an intra-venous route, the acute LD_50_ in rats was observed as 29 mg/kg of sanguinarine [34]. Kosina et al. and Psotova et al. attributed the toxicity of sanguinarine to a higher dose of sanguinarine administration via the intraperitoneal route [16]. However, more detailed assays need to be performed on sanguinarine’s toxicity and other physiological variables.

## 4. Materials and Methods

### 4.1. Protein Preparation

The human PPARα–LBD (NM_001001928, residues 196–468) was expressed as N-terminal 6×His fusion protein from the expression vector pET24a (Novagen, Darmstadt, Germany). The BL21 (DE3) cells transformed by expression plasmids were grown in LB broth at 25 °C to an OD_600_ of ~1.0, and induced with 0.1 mM isopropyl 1-thio-β-D-galactopyranoside (IPTG) at 16 °C for 14–16 h. Cells were harvested and sonicated in 200 mL of extraction buffer (20 mM Tris pH 8.0, 150 mM NaCl, 10% glycerol, and 25 mM imidazole) per 6 L of cells. The lysate was centrifuged at 20,000 rpm for 30 min, and the supernatant was loaded on a 5 mL Ni-loaded HiTrap HP column (GE Healthcare). The column was washed with extraction buffer, and the protein was eluted with a gradient of 25–500 mM imidazole. The PPARα–LBD was further purified by gel filtration using a HiLoad 26/600 Superdex 200 column (GE Healthcare, Pittsburgh, PA, USA) (elution buffer, 25 mM Tris-HCl (pH 7.5), 100 mM NaCl, 2 mM DTT). To prepare the protein–ligand complex, we added a five-fold molar excess of sanguinarine to the purified protein, followed by a filter concentration to 10 mg/mL. The PPARα–LBD was complexed with two-fold molar of a SRC1-2 peptide (RHKILHRLLQEGSP) before filter concentration.

### 4.2. Coactivator Binding Assays

The binding of the various co-factor peptide motifs to the PPAR–LBDs (PPARα, PPARδ, and PPARγ) in response to ligands was determined by AlphaScreen assays using a hexahistidine detection kit from Perkins-Elmer (Norwalk, CT, USA) as described before [21,22,36]. Compounds were prepared in 96-well plates. The mixture was then added, comprised of approximately 20–40 nM PPAR LBDs and 20 nM biotinylated co-factor peptides, in the presence of 5 µg/mL streptavidin donor and nickel chelate acceptor beads in a buffer that contained 50 mM MOPS, 50 mM NaF, 0.05 mM CHAPS, and 0.1 mg/mL bovine serum albumin, all adjusted to a pH of 7.4. A luminescence signal was detected by Perkins-Elmer multimode microplate reader. The peptides with an N-terminal biotinylation are listed below:

SRC1-2, SPSSHSSLTERHKILHRLLQEGSP;

SRC2-3, QEPVSPKKKENALLRYLLDKDDTKD;

SRC3-3, PDAASKHKQLSELLRGGSG;

NCoR-1, QVPRTHRLITLADHICQIITQDFAR;

SMRT-2, ASTNMGLEAIIRKALMGKYDQ.

### 4.3. Transient Transfection Assays

The HEK–293T cells were maintained in DMEM containing 10% fetal bovine serum, and were transiently transfected using Lipofectamine 2000 (Invitrogen). All mutant PPARα and PPARδ plasmids were created using the Quick-Change site-directed mutagenesis kit (Stratagene, La Jolla, CA, USA). The resulting plasmids were confirmed by DNA sequencing. Before 24 h of transfection, 24-well plates were plated (5 × 10^4^ cells per well). For the nuclear receptor luciferase reporter assay, the cells were transfected with 200 ng Gal4-LBDs of various nuclear receptors and 200 ng of pG5Luc reporter (which contained five GAL4 binding sites) (Promega, Madison, WI, USA) [37,38]. For native promoter-reporter assays, the cells were co-transfected with plasmids encoding full-length PPARs and a peroxisome proliferator hormone response element (PPRE). Ligands were added 5 h after transfection. Cells were harvested 24 h later for the luciferase assays with the dual-luciferase reporter assay system (Promega, Madison, WI, USA). The luciferase activities were normalized to renilla activity, co-transfected as an internal control. The dose curves were fitted by GraphPad Prism 8.

### 4.4. Thermal Shift Assay

Thermal shift assay (TSA) was performed using 20 μL experimental unit containing 10 μM PPARs–LBD, two-fold molar compounds or five-fold molar co-activator peptide SRC1, and 2.5× SYPRO Orange (Sigma) in a buffer containing 150 mM NaCl, 25 mM Tris, and 5 mM dithiothreitol (DTT), at pH 7.5. The samples were heated from 30 to 80 °C at a rate of 1 °C per 5 s, and the fluorescence data were obtained on a CFX96 Real-Time PCR System (Bio-Rad, Hercules, CA, USA). Half of the maximum temperature (Tm) value of proteins was calculated by GraphPad Prism 8 and fitted using Boltzmann sigmoid curves.

### 4.5. Preparation of Mouse Primary Hepatocytes

Mouse hepatocytes were prepared according to a previously described procedure [39]. C57/BL/6 male mice (6–8 weeks old, Beijing Vital River Laboratory Animal Technology Co., Ltd., Beijing, China) were intraperitoneally anesthetized with Nembutal, and the liver was perfused with a liver perfusion medium, followed by a liver digestion medium. Hepatocytes were dispersed in a hepatocyte wash medium by dissection and gentle shaking. After filtration through a 100-μm nylon mesh filter, the hepatocytes were isolated by repeated centrifugation at 50× *g* for 3 min. The isolated hepatocytes were cultured in growth media containing 10% FBS in DMEM medium, with type 1 collagen-coated 12-well plates (Iwaki, Tokyo, Japan) at a cell density of 2 × 105 cells/well. After incubation for 12 h at 37 °C in a 5% CO_2_ atmosphere, the hepatocytes were treated with fenofibrate or sanguinarine for 48 h.

### 4.6. 3T3-L1 Cells Differentiation

The 3T3–L1 cells were maintained in growth media containing 10% FBS in a DMEM medium. For adipocyte differentiation, two days after cells reached confluency, they were cultured in the DMEM growth medium, supplemented by 1 μM dexamethasone (TagerMol, Shanghai, China), 0.5 mM IBMX (TagerMol, Shanghai, China), and 10 μg/mL insulin (TagerMol, Shanghai, China), for 48 h. Cells were maintained in the DMEM growth medium, containing 10 μg/mL insulin, for an additional 48 h. Following this, the medium was replaced by a growth medium, and this growth medium was replaced every 48 h. For ligand-induced adipocyte differentiation, cells were treated with 1 µM rosiglitazone or sanguinarine 2 days after confluence. Cells were exposed to ligands constantly for 7 days until fat cells were observed. The cells were then stained with a filtered oil red O stock solution (0.5 g of oil red O (Sigma, StLouis, MO, USA) in 100 mL of isopropyl alcohol) for 15 min at room temperature.

### 4.7. Quantitative Real-Time PCR (qPCR)

Total RNA was extracted from hepatocytes and 3T3–L1 cells with a Trizol reagent (Sigma, StLouis, MO, USA). The RNA was reverse-transcribed using the TAKARA reverse-transcription kit. Real-time quantitative PCR was performed with Hieff qPCR SYBR Green Master Mix (Yeasen Biotech, Shanghai, China) using a CFX96 Real-Time PCR Detection System (Bio-Rad, Hercules, CA, USA). The primer sequences of all genes were reported beforehand [40,41]. The mRNA expression was normalized to GAPDH, or 36B4.

### 4.8. Crystallization and Structure Determination

The crystal of the PPARα–sanguinarine–SRC1-4 complex was grown at room-temperature in hanging drops, containing 1.0 μL of the ligand-protein solutions and 1.0 μL of the well buffer, itself containing 0.2 M ammonium formate, and 20% (*w*/*v*) polyethylene glycol 3350. The crystals were directly flash-frozen in liquid nitrogen for data collection. Diffraction data were collected at beamline BL17U1 of the Shanghai Synchrotron Radiation Source. The observed reflections were reduced, merged, and scaled with DENZO and SCALEPACK, in the HKL2000 package [42]. The search model for PPARα–sanguinarine was 2P54 in the Protein Data Bank. The structure was determined by molecular replacement in the CCP4 suite [43]. Manual model building was carried out with Coot [44], followed by Refmac5 refinement in the CCP4 suite [45]. Structural figures were made with PyMOL (version 2.3.3, Schrödinger, New York, NY, USA). The structure of PPARα–sanguinarine was deposited in the PDB, under the code 7C6Q.

## 5. Conclusions

In our study, we identified sanguinarine as a dual agonist of PPARα/γ by biochemical and cell-based promoter-reporter gene assays. Moreover, sanguinarine modulates the key PPARα-target gene expression in hepatocytes and the key PPARγ-target gene expression in adipocyte differentiation, yet shows a lower adipogenic activity than TZDs. We solved the crystal structure of sanguinarine that is bound to PPARα, which provided resolute evidence of the direct binding of sanguinarine with PPARα. In addition, structural and functional analysis revealed that sanguinarine specifically binds to PPARα through its unique structural features, distinct from classic, Y-shaped binding pockets of PPARα ligands. Our study further explains why sanguinarine selectively binds to PPARα and PPARγ, rather than PPARδ subtypes. Overall, the molecular basis for the selectivity of sanguinarine binding to PPARα/γ, and the unique structural mechanism of sanguinarine binding to PPARα, collectively provide a new strategy for the design of dual-targeting PPARs ligands.

## Data Availability

The structure of the PPARα–sanguinarine–SRC1 ternary complex was deposited to the Protein Data Bank with PDB ID 7C6Q.

## Supplementary Materials

Figure S1: Chemical structures of dual PPARα/γ agonists. Figure S2: The binding of the biotinylated SRC1-2 peptide to PPARs–LBD, in response to ligands from the Enzo Natural Compound Library (502 compounds), was determined by AlphaScreen™ assays. Figure S3: Sequence alignment of human PPARα–LBD with human PPARδ– and PPARγ–LBDs. Figure S4: Chemical structures of dual PPARα/γ agonists. Table S1: Data collection and refinement statistics.

## Sample Availability

Samples of the compounds are not available from the authors.

## Abbreviations

PPAR	Peroxisome proliferator-activated receptors
AF-1	activation function 1
DBD	DNA-binding domain
LBD	ligand-binding domain
NASH	nonalcoholic steatohepatitis
TZDs	Thiazolidinediones
TSA	thermal shift assay
