# Supplementary material for: Structural Basis for PPARs Activation by The Dual PPARα/γ Agonist Sanguinarine: A Unique Mode of Ligand Recognition
Source: Molecules. 2021 Oct 3;26(19):6012. doi: 10.3390/molecules26196012 (PMC8512631; doi:10.3390/molecules26196012)
Supplement: Supplementary file 1 [file molecules-26-06012-s001.zip › molecules-1386290-supplementary.pdf]

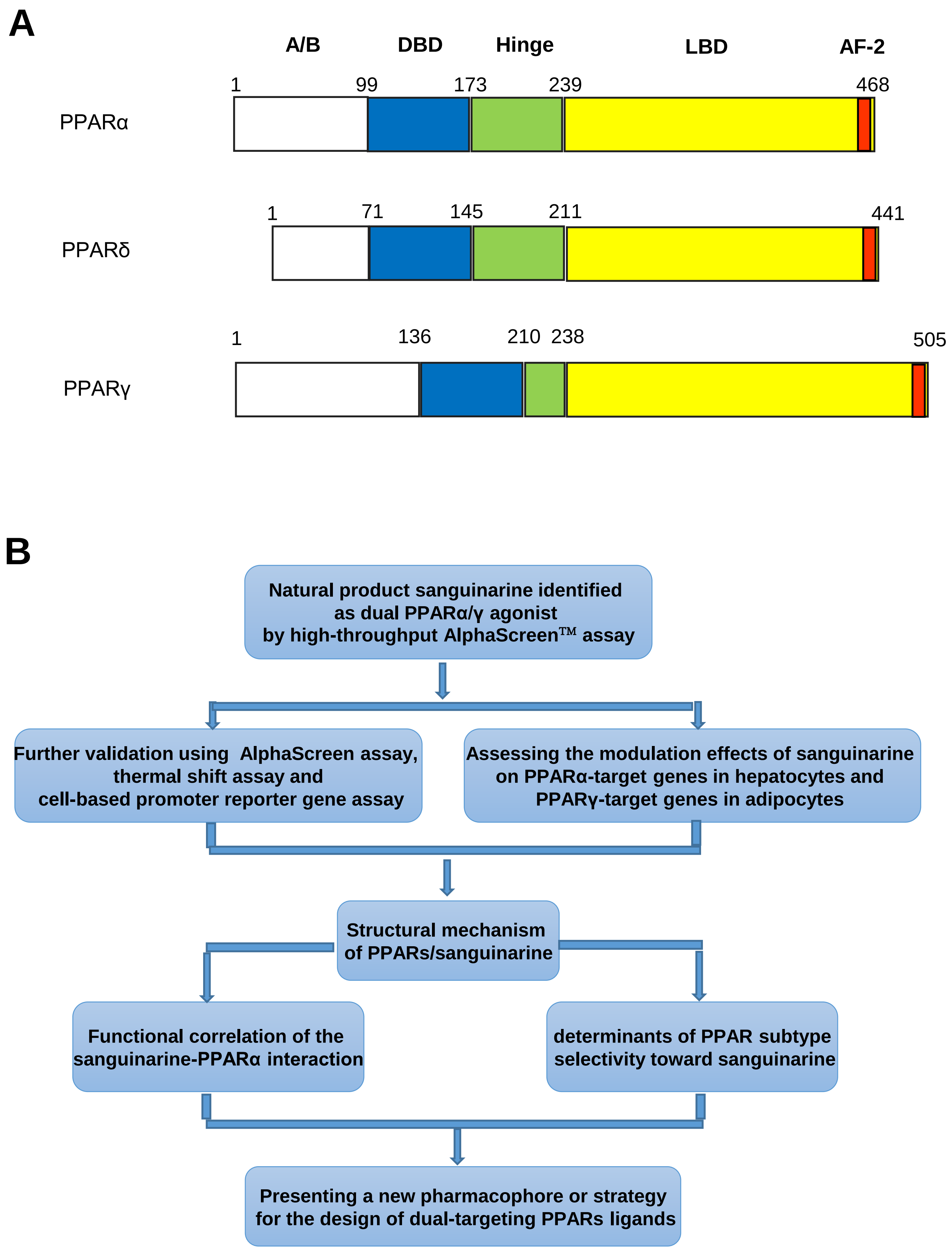

Figure S1. (A) Schematic diagram showing functional domain of PPARs. **(B)** General workflow overview.

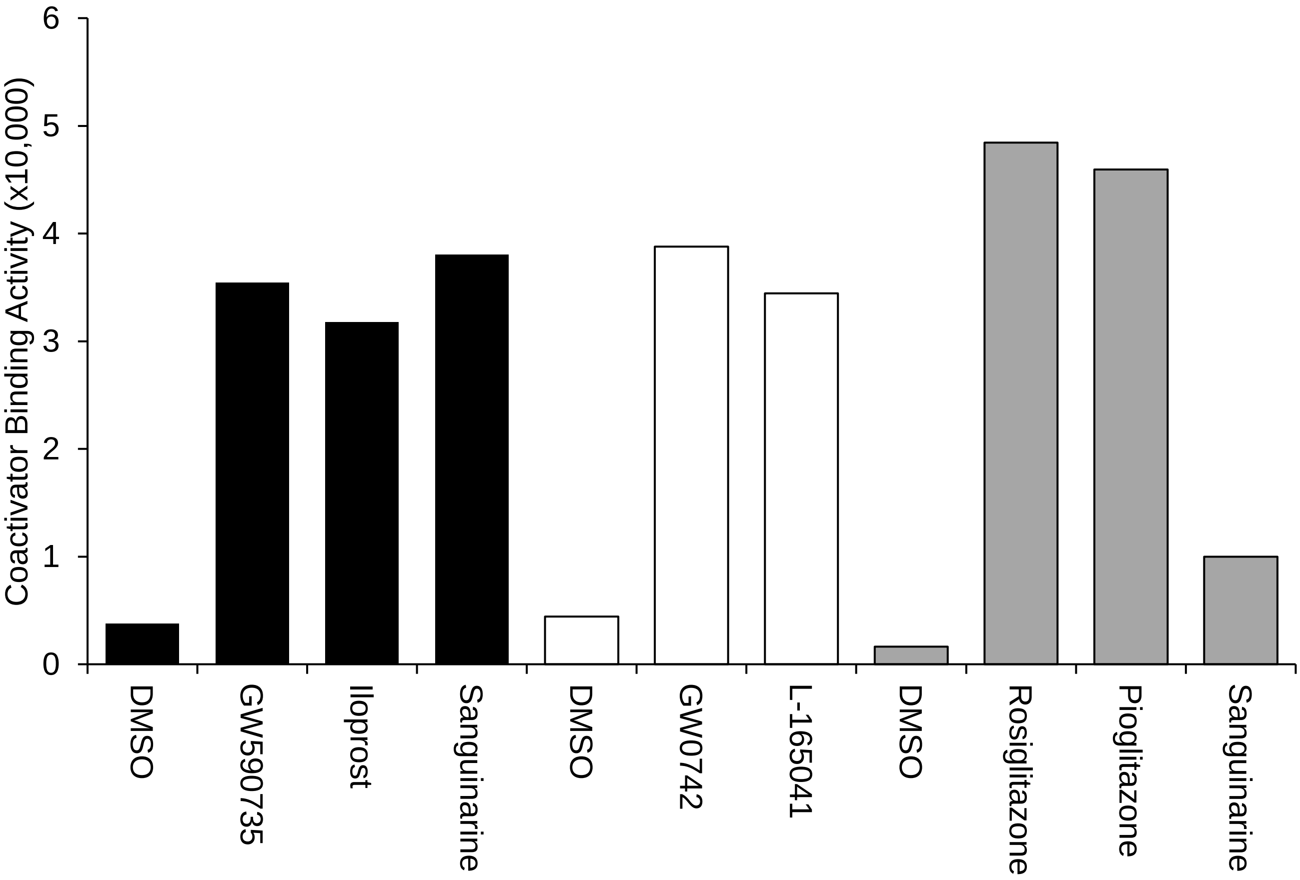

Figure S2. The binding of the biotinylated SRC1-2 peptide to PPARs LBD in response to ligands from the Enzo Natural Compound library (502 compounds) was determined by AlphaScreen™ assays. The experiments were conducted with approximately 20–40 nM receptor LBD and 20 nM biotinylated co-factor peptides in the presence of 5 μM ligands. The compounds that induced more than 2-fold co-activator binding activity of PPARs LBD are shown. PPARα, black bars; PPARδ, white bars; PPARγ, gray bars.

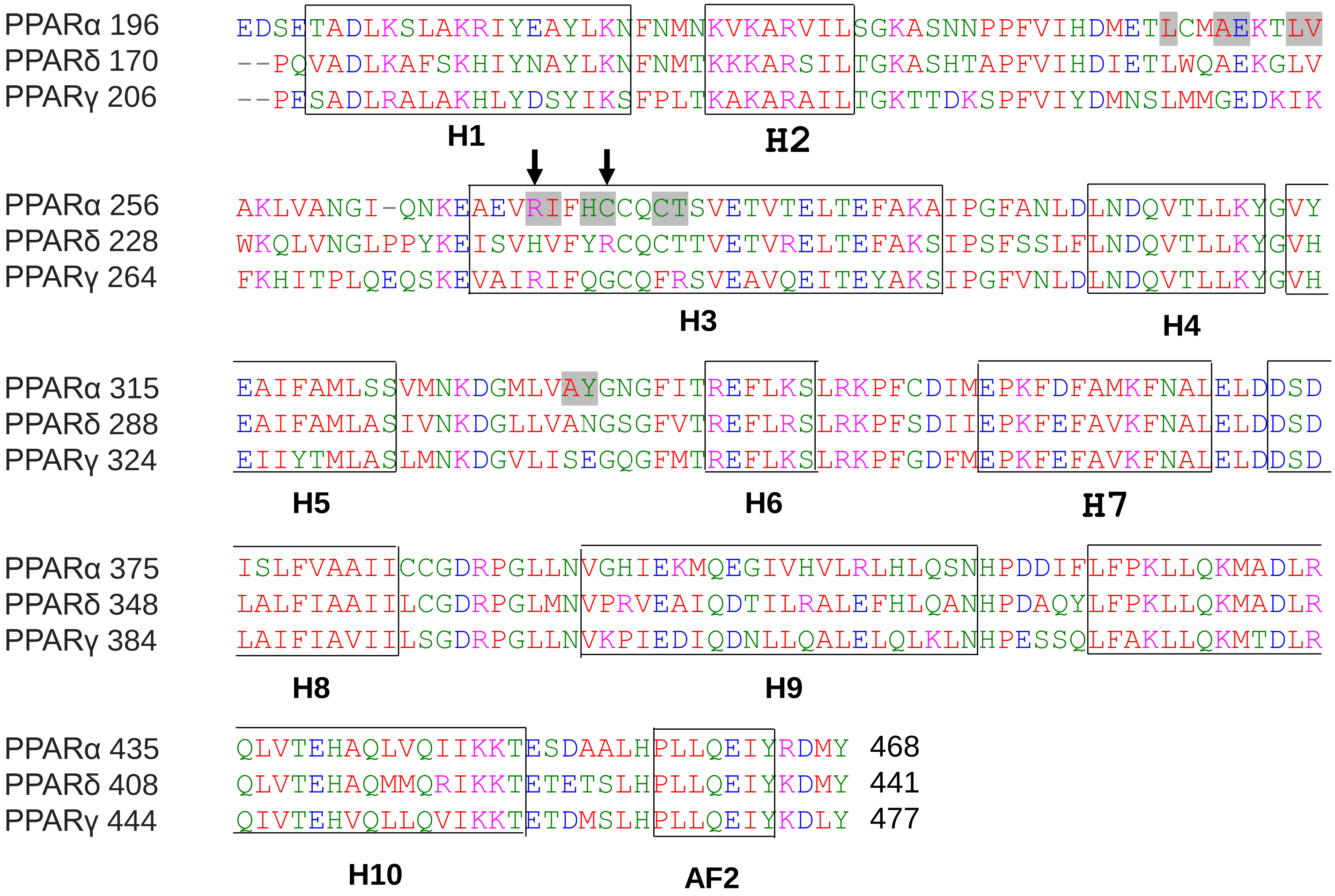

Figure S3. Sequence alignment of human PPARα LBD with human PPARδ and PPARγ LBDs. The secondary structural elements are boxed and annotated below the sequences with residues colored according to their sidechain property. Residues with small and hydrophobic sidechains are in red, acidic sidechains in blue, basic sidechains in magenta, and hydroxyl and amide sidechains in green. And the PPARα residues that interact with sanguinarine are shaded in gray, the residues that determine the sanguinarine binding selectivity are noted with arrows.

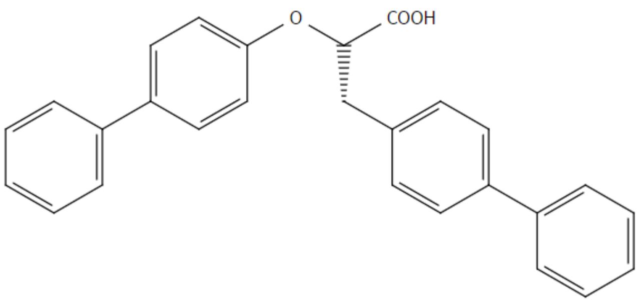

LJ 570

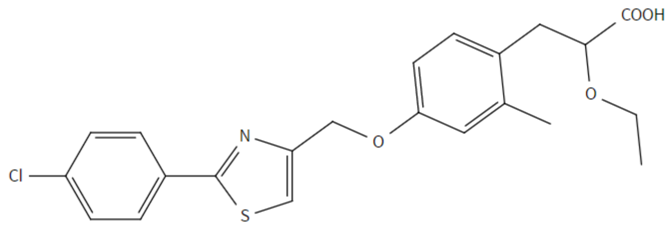

24

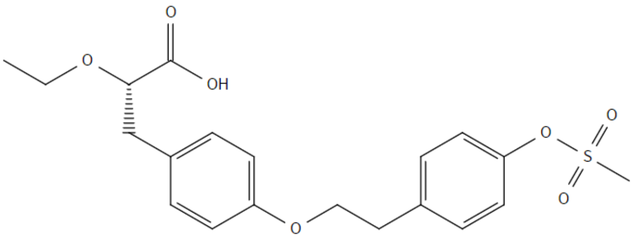

Tesaglitazar

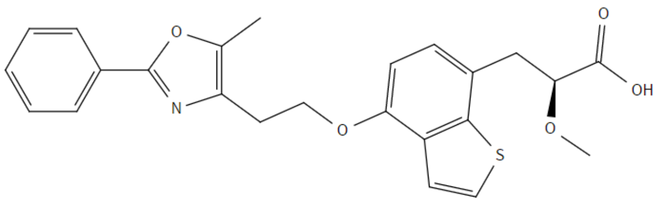

Aleglitazar

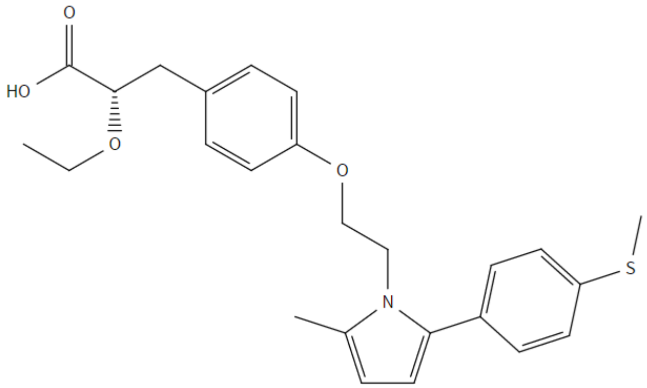

Saroglitazar

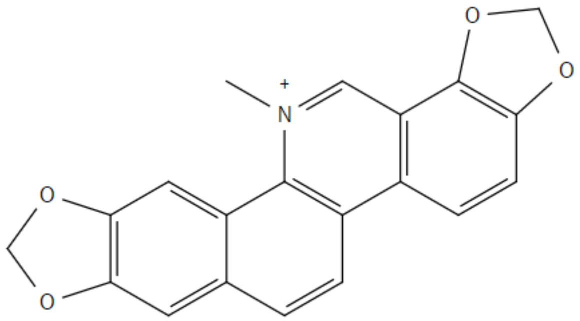

Sanguinarine

Figure S4. Chemical structures of dual PPARα/γ agonists.

Table S1. Data collection and refinement statistics

**Parameters**	**PPARα/Sanguinarine/SRC1-2**
**Data collection**	
Space group	P212121
Cell dimensions	
a,b,c (Å)	45.55 61.50 98.48
α,β,γ(°)	90.000 90.000 90.000
Resolution(Å)	44.26-2.76(2.91-2.76)[1]
Rmerge[2]	0.116(0.424)
I/σ	13.4(5.8)
Completeness (%)	99.9(100)
Redundancy	7.0(7.3)
**Refinement**	
Resolution (Å)	44.30-2.76
No.reflections	7786
Rwork[3]/Rfree[4] %	19.09/26.07
No.atoms	
Protein	2153
Ligand/ion	25
Water	53
B factors ($Å^2$)	
Protein	45.12
Ligand/ion	34.90
Water	35.97
R.M.S.D	
Bond lengths (Å)	0.0065
Bond angles (°)	1.4349

1 Values in parenthesis are for highest-resolution shell.

2 $R_{merge}=\sum_{hkl}\sum_i |I_i - \langle I \rangle| / \sum_{hkl}\sum_i I_i$ where I_i is the intensity of a reflection i of the group represented by the unique reflection hkl, and $\langle I \rangle$ is the average intensity of the group represented by reflection hkl.

3 $R_{work}=\sum|F_{obs} - F_{calc}|/\sum|F_{obs}|$ where F_{obs} and F_{calc} are the observed and calculated structure factor amplitudes, respectively.

4 R_{free} is calculated using the same equation as that for R_{work} but 5% of reflections were chosen randomly and omitted from the refinement.
